# Unusual Case of a Traumatic Proximal Humeral Physeal Fracture in an Adolescent

**DOI:** 10.7759/cureus.74504

**Published:** 2024-11-26

**Authors:** Gustavo Tafoya-Arreguín, Veronica Villalobos-Salazar, Héctor Haro-Gómez, Jaime Ruelas-Pérez, José de Jesús Martínez-Ruíz

**Affiliations:** 1 Surgery, Centro Universitario de Ciencias de la Salud, Universidad de Guadalajara, Guadalajara, MEX; 2 Traumatology and Orthopaedics, Hospital Civil Antiguo de Guadalajara "Fray Antonio Alcalde", Guadalajara, MEX; 3 Surgery, Centro Universitario de Tonala, Universidad de Guadalajara, Guadalajara, MEX; 4 Plastic and Reconstructive Surgery, Instituto Mexicano del Seguro Social, Unidad Médica de Alta Especialidad, Hospital de Traumatología y Ortopedia, Monterrey, MEX

**Keywords:** adolescent, fracture, physeal injury, proximal humerus, trauma

## Abstract

Shoulder injuries, specifically proximal humeral fractures, are uncommon in skeletally immature patients. The anatomic characteristics of the humerus are determined by its ossification development, which is quite particular in the proximal segment where four principal segments have been outlined as fracture components in adults. Hereby, we present the case of an 18-year-old female who suffered a traffic accident that elicited a proximal humeral injury through the physeal line of the anatomical neck, as well as the treatment given and her clinical outcome with an effort to highlight the complexity of the diagnosis and management of this injury given the transitional bone age.

## Introduction

Pediatric shoulder trauma is a rather rare query in the emergency department; lesions that require surgical management are even more uncommon [1]. Fractures that affect the proximal humeral epiphysis account for about 2%-3% of all physeal injuries [2]. The magnitude and direction of the applied force will originate certain fracture patterns along with the location and maturity of the growth center. Physis that are near to the closure will fracture differently than those that are fully open [3]. 

Adolescents are considered the age group with the highest rate of physeal injury; even though the physis becomes thinner as it reaches skeletal maturity, the activity level of a person and size itself can outmost the previously alleged advantage [3]. Therefore, skeletally immature patients are at increased vulnerability to injuries that are distinct to the pediatric and adult populations [4]. 

The distinctive proximal humeral physis is the result of the coalescence of three secondary centers of ossification, which occurs generally at the age of five to seven years old [1]. At birth, this growth plate is flat and transverse, but around the fourth month of age, it becomes progressively tented; its apex is situated somewhat centrally in the anterior segment and more laterally in the posterior humerus [5]. The epiphyseal ossification center appears at six months of age, while the greater and lesser tuberosity centers emerge more or less at three and five years, respectively [1]. Closure of the open proximal humeral physis starts at about 14 years of age at the center and ceases at approximately 17 years of age, the posterolateral portion being the last to fuse [5]. 

Apart from an accurate radiologic assessment that should include documentation of signs of disruption and confirmation of normal anatomic relationships, a CT scan is useful in the evaluation of extraordinarily complex fractures or for purposes such as preoperative planning [2]. 

In the setting of proximal humeral physis fractures, most are handled successfully nonoperatively. The exception is the older child near skeletal maturity with a severely displaced fracture [1], a controversial scenario. Management, regardless of age, aims to realign the physis and restore joint congruity [2]. 

In the study of Abbot et al. (2024), 125 pediatric patients who had sustained proximal humeral fractures (16% were physeal injuries) and were treated operatively vs. nonoperatively were evaluated for the follow-up with the Quick Disabilities of the Arm, Shoulder, and Hand (DASH) questionnaire. Scores that dictated poorer clinical outcomes were associated with physeal fractures and age older than 12 years [6]. Extensive follow-up visits can improve results; therefore, clinical evaluation at least three to six months succeeding fracture management is recommended to confirm restoration of growth plate function; if any setback is noted, prompt action can be performed [2]. 

The intention of this work is to present a clinical case along with a literature review of proximal humerus growth plate injuries, their diagnosis, and management, and to discuss which of them apply to this unusual case where the mechanism is unclear, diagnosis is not obvious, and management is debatable, in the setting of a patient that has a transitional bone age maturation.

## Case presentation

We present the case of an 18-year-old female who sustained a motorcycle accident on a high-speed road. She was first taken to a primary center where they stabilized and provided her with first aid; after that, she was remitted to our tertiary trauma center because of an injury in her right shoulder. 

**Figure 1:**
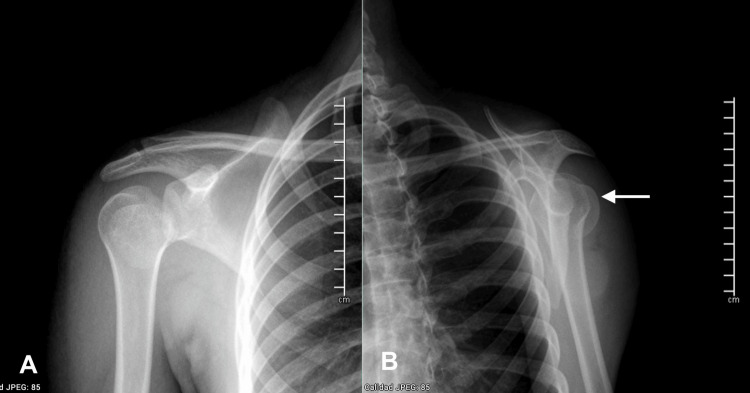
Initial anteroposterior (A) and axial (B) radiographs The white arrow indicates a bone fragment in the posterior situation.

Initial clinical evaluation elicited pain and limitation in all the right shoulder range of motion, although no deformities were noted, and the neurovascular exam showed no abnormalities. Thoracic and pelvis X-rays were taken to dismiss axial injuries and shoulder radiographs were taken. Whilst the anteroposterior view displayed apparent articular congruency and no clear osseous injuries; the scapular view revealed a posteriorly located bone fragment; therefore, a conclusive proximal humeral fracture (Association of Osteosynthesis (AO) 11-E/3.1) was diagnosed (Figures 1A-1B).

**Figure 2:**
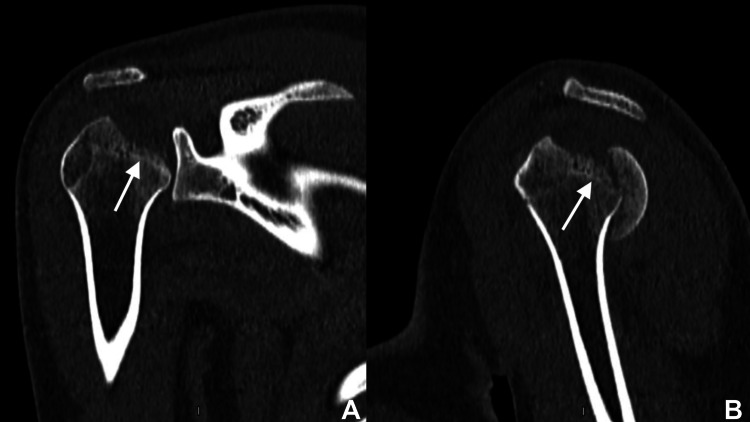
Initial CT showing the coronal (A) and sagittal (B) slides The white arrows signal a fracture line through the physeal scar of the proximal humerus.

**Figure 3:**
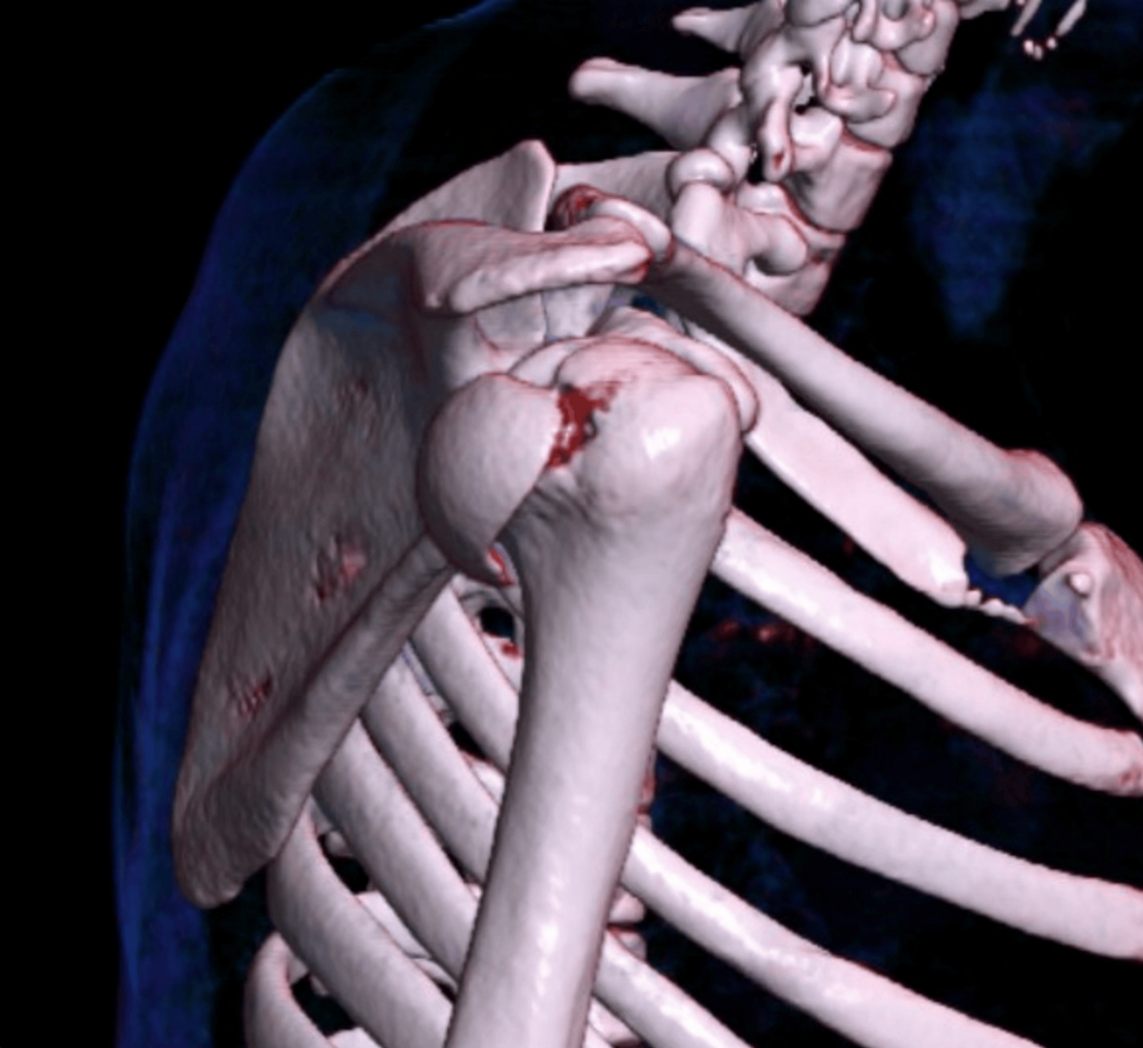
Initial 3D CT reconstruction

A CT scan with 3D reconstruction was obtained to understand the fracture pattern fully and for surgical planning. Coronal and sagittal slides confirmed a humeral head fracture, with a single articular bone fragment posterior to the glenoid cavity. A 3D reconstruction not only confirms the posterior location of the bone fragment but also helps define the fracture line contours of the physeal scar of the anatomical neck of the proximal humerus (Figures 2-3). 

**Figure 4:**
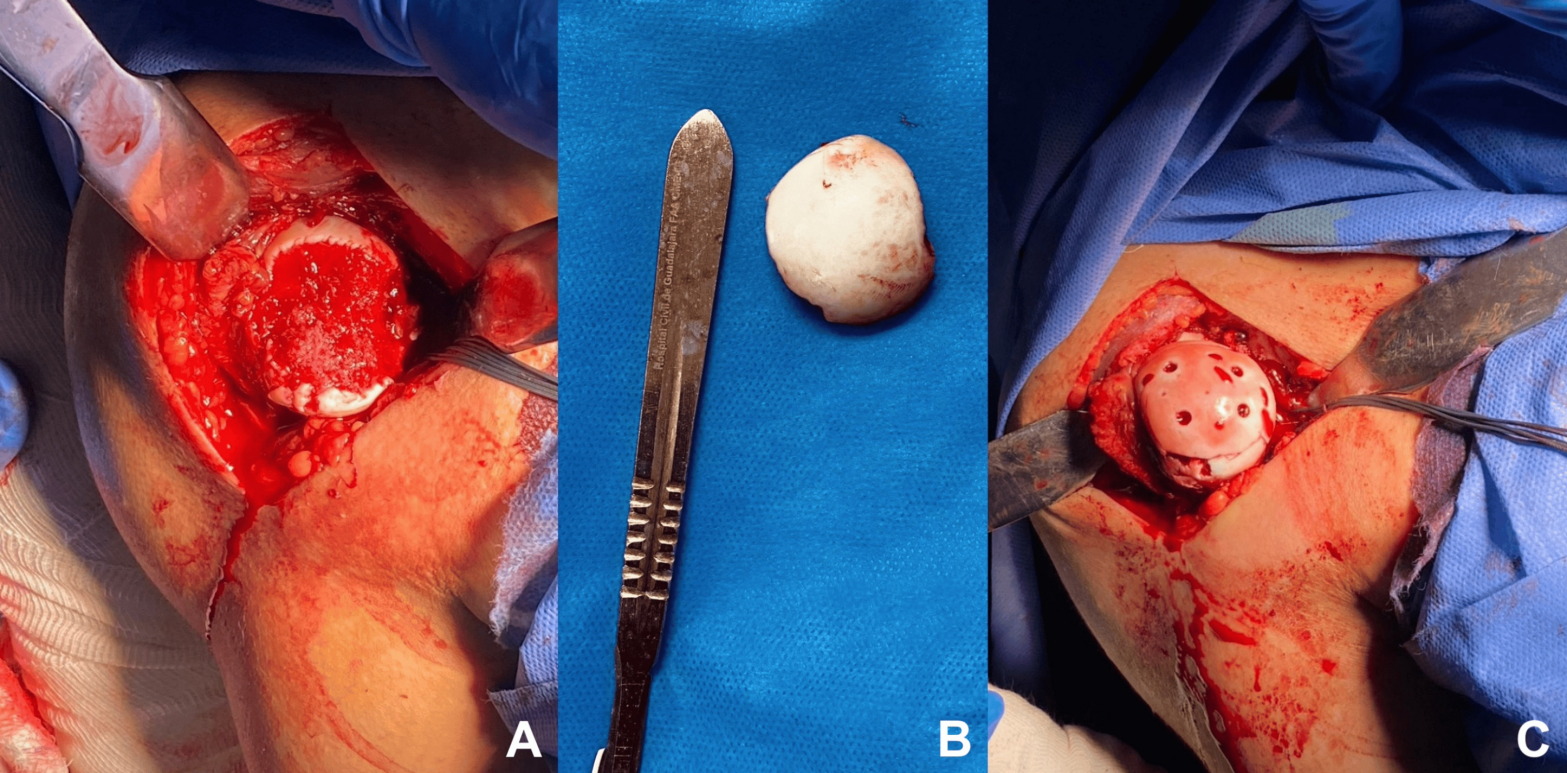
Surgical approach (A), bone fragment (B) and definitive fixation (C) In Figure 4B, a bone fragment is placed beside a number four scalpel for referenced dimensions.

**Figure 5:**
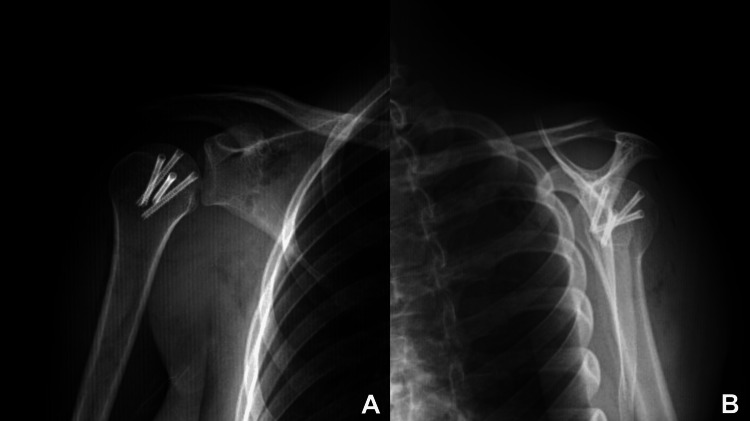
Immediate postoperative anteroposterior (A) and axial (B) radiographs

Surgical management was decided and comprised an open reduction through the deltopectoral approach, with a subsequent subscapularis tenotomy and vertical capsulotomy. By gentle traction and maximal external rotation of the arm, the posteriorly located bone fragment devoid of any soft tissue attachment was recovered, anatomic reduction and temporary fixation with threaded Kirschner wires (k-wires) was executed, and definitive fixation with four acephalic 2.7 mm screws (Acutrack, Acumed LLC, Hillsboro, OR, USA) in a quadrangular configuration from medial to lateral until totally buried below articular cartilage was performed (Figures 4A-4C). Capsule and subscapularis tendon repair was done using non-absorbable sutures, ensuring stability in all ranges of motion. A standard surgical dressing was placed in the final wound. Immediate postoperative radiographs showed congruent and stable fixation (Figures 5A-5B). 

The patient was kept with a sling until suture retirement two weeks later; after that, she began mobilization with pendulum exercises until four weeks postop when passive movement was initiated and progressed to active motion. Socioeconomic issues made it difficult for the patient to comply with a physical therapy program, although it was early indicated. 

**Figure 6:**
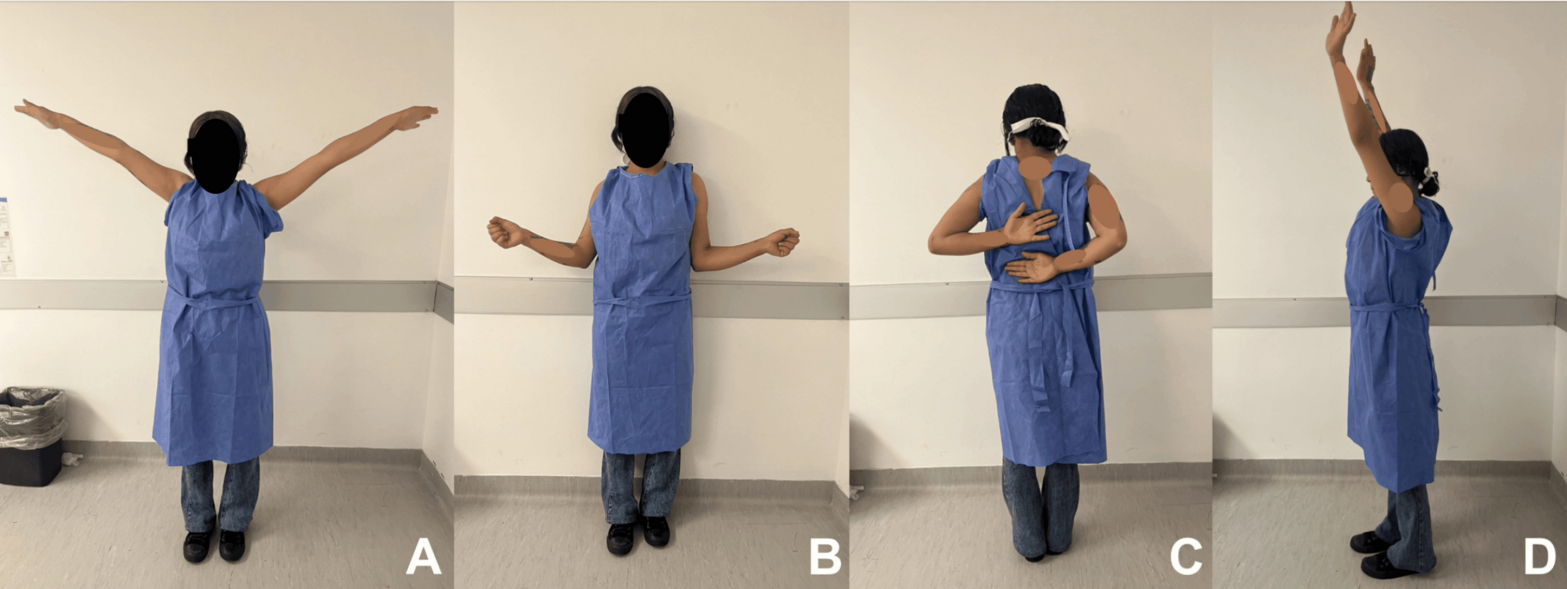
Clinical evaluation at the one-year follow-up visit: abduction (A), external rotation (B), internal rotation (C), and frontal flexion (D)

**Figure 7:**
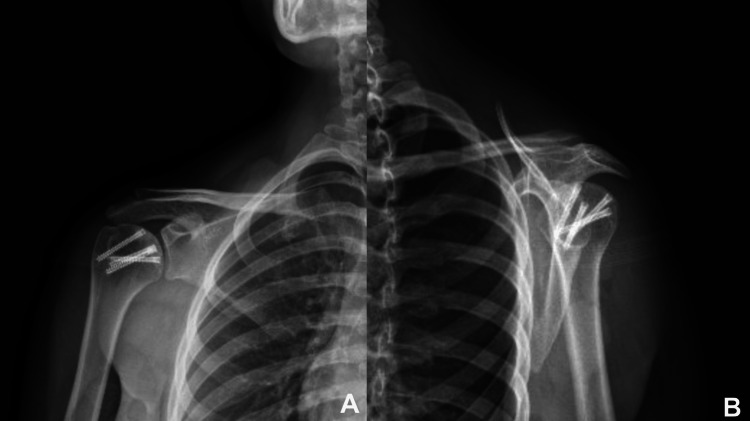
Follow up anteroposterior (A) and axial (B) radiographs No radiologic signs of arthrosis are evident.

**Figure 8:**
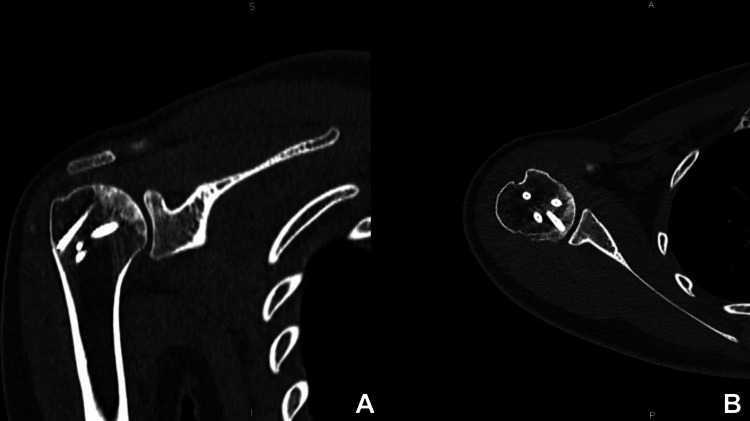
Follow up CT: coronal (A) and axial (B) slides The CT shows adequate bone consolidation at the one-year follow-up visit.

**Figure 9:**
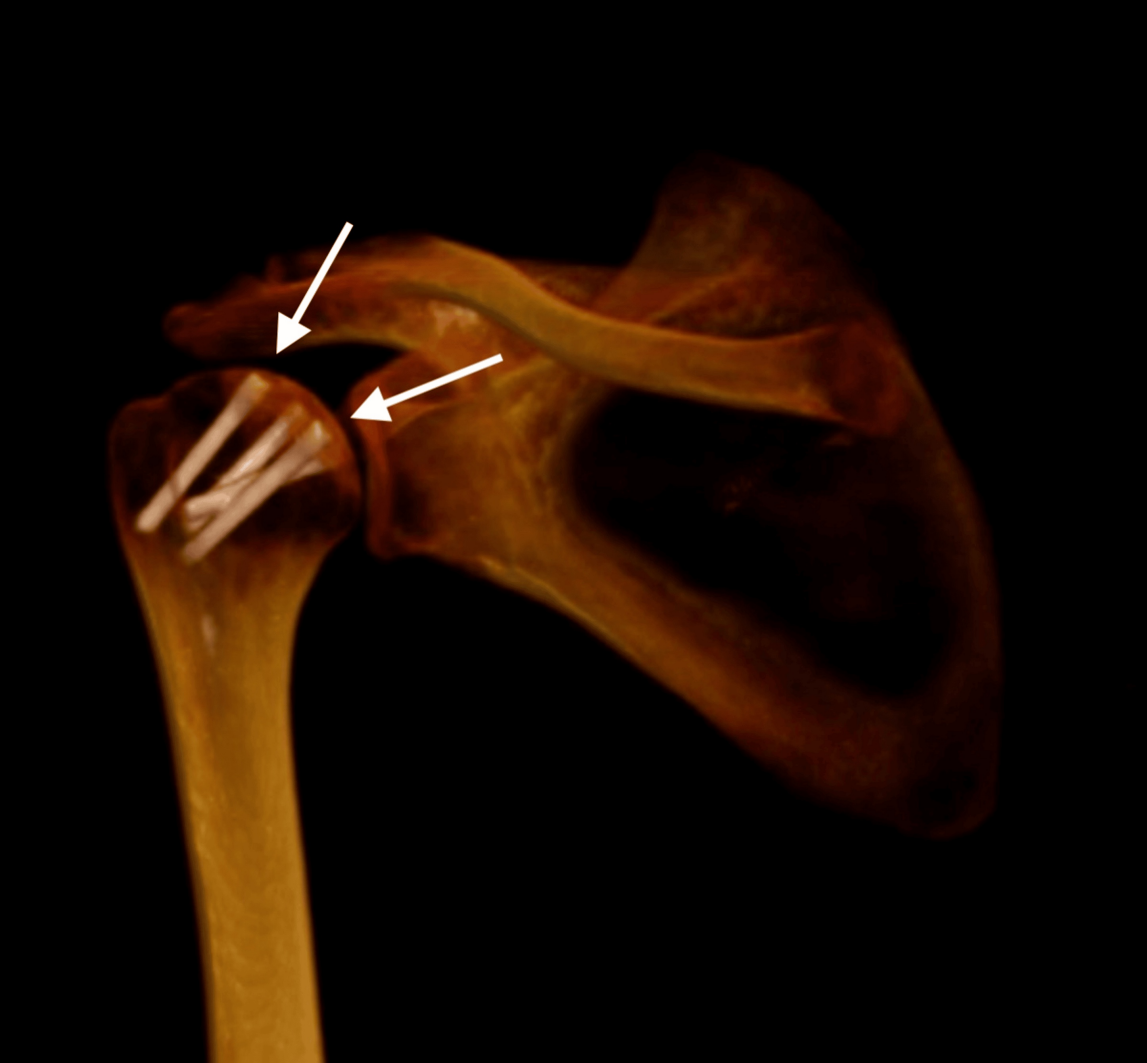
A 3D metal suppression CT reconstruction at the one-year follow-up visit The white arrows signal adequate subchondral localization of the screws.

The last follow-up was made one year postoperatively. At the moment, she has fully reincorporated herself into her work (as a laborer at a berry farm), with no impairment in social activities. Clinical evaluation revealed limitations for abduction and external rotation regarding the contralateral extremity (130º/180º and 30º/45º) (Figures 6A-6D). She referred to mild discomfort only after long hours of physical activities. The Constant Score indicated a good shoulder function overall (71 points). Radiographs and CT showed satisfactory bone consolidation, without clear signs of osteonecrosis and adequate articular congruency; no hints of arthrosis were found (Figures 7-9). 

## Discussion

A recurrent dilemma appears when attending adolescent patients, as their injuries are not equal to those of children or adults, and we cannot expect the most common scenarios. Hence, we highlight the rarity of this proximal humeral injury: if we were talking about an adult patient, a single clear fracture line is not usual, but chronological age is not consistent with an open growth center injury. Yet the isolated anatomical neck fracture of our patient is consistent with a physeal fracture, and given that physis are more resistant to compressive forces or traction than to torsion and shear ones [3], we suspect a dislocation or subluxation happened at some point of the accident. 

Diagnosis of a physeal fracture is not always easy; age and mechanism of injury are often key [4]. In the presented case, both aspects of the patient are not the most seen to proceed with general guidelines of diagnosis and management, given the bone developmental phase and the unclear biomechanics of the injury in the setting of a high-speed motor accident. 

Initial radiographs were insufficient to fully understand this lesion; therefore, we used CT with 3D reconstruction, which also helped surgical planification from approach to intraoperative decisions. We do not dismiss that an MRI might have been useful in providing insight into the extent and pattern of physeal injury [7], but resources were not available. 

As aforementioned, we were not upon a common fracture; definitive literature on the optimal management of an articular injury of this kind was not found, but we did encounter anatomical and biomechanical similarities to a Delbet I fracture in the proximal femur; therefore, we opted for the described open reduction and internal fixation (ORIF) procedure. 

A one-year follow-up is presented, with X-rays and CT that show survival of the bone fragment and adequate secondary consolidation of the fracture thanks to the stable fixation obtained; in this case, the main concern was not a growth disturbance but osteonecrosis [8,9] of the fragment, which until now can be discarded but should be kept constantly evaluated; the remaining concern is the development of post-traumatic arthrosis; therefore, continuous follow-up is mandatory. Clinical evaluation shows good recovery of all ranges of motion, with restriction of external rotation and abduction that does not impair daily activities. 

## Conclusions

This case shows a rare presentation of a proximal humeral shear injury closely related to the physeal scar, which held an interesting discussion from diagnosis to treatment because the history of the mechanism of injury was not available, general management guidelines were not suitable to follow, and literature referral was not found in our search. Aside from successful radiological appearance, the greatest achievement is clinical evolution, given our patient has fully reintegrated into her daily activities with no pain or limitations. 

We believe that more often than physicians care to admit, certain traumatic injuries of the shoulder can come up as a diagnosis and management predicament. The importance of reporting this case resides not only in the rarity of the shearing pattern and its close relation to the physeal plate because it is possible that there are other patients who had suffered it, but information about management and outcomes experienced is not abundant, and should be available to the medical community.
